# Revealing region-specific biofilm viscoelastic properties by means of a micro-rheological approach

**DOI:** 10.1038/s41522-016-0005-y

**Published:** 2016-12-05

**Authors:** Huayu Cao, Olivier Habimana, Ashkan Safari, Rory Heffernan, Yihong Dai, Eoin Casey

**Affiliations:** 1 School of Chemical and Bioprocess Engineering, University College Dublin (UCD), Belfield, Dublin 4, Ireland; 2 School of Biological Sciences, The University of Hong Kong, Hong Kong, PR China

## Abstract

Particle-tracking microrheology is an *in situ* technique that allows quantification of biofilm material properties. It overcomes the limitations of alternative techniques such as bulk rheology or force spectroscopy by providing data on region specific material properties at any required biofilm location and can be combined with confocal microscopy and associated structural analysis. This article describes single particle tracking microrheology combined with confocal laser scanning microscopy to resolve the biofilm structure in 3 dimensions and calculate the creep compliances locally. Samples were analysed from *Pseudomonas fluorescens* biofilms that were cultivated over two timescales (24 h and 48 h) and alternate ionic conditions (with and without calcium chloride supplementation). The region-based creep compliance analysis showed that the creep compliance of biofilm void zones is the primary contributor to biofilm mechanical properties, contributing to the overall viscoelastic character.

## Analytics: A better look at biofilms

Combining two distinct techniques allows analysis of the structure and physical properties of bacterial biofilms in fine detail. Eoin Casey and colleagues, from University College Dublin, Ireland, and the University of Hong Kong, applied a combined non-destructive procedure to cultured biofilms, for determining biofilm slimy properties and structural features. Tracking the movement of tiny fluorescent beads embedded in the biofilms yielded insights into their localised material properties. The same samples were also analysed using a laser-based imaging technique called confocal microscopy, which added additional structural insight to the biofilm samples. This combination allowed an improved regional understanding of the biofilms’ varying material and mechanical properties, including viscosity and elasticity. In future, the researchers hope to add chemical analysis into the mix, producing a method to search for better ways to treat and eradicate biofilms.

## Introduction

The characterisation of biofilms in terms of their material properties is important for the fundamental understanding of methods for the control of unwanted biofilms.^1, 2^ However comprehensive quantification of the mechanical properties of biofilm remains a major challenge, in part due to the temporal, spatial and compositional dynamics of the extracellular matrix,^3^ but also due to the lack of available techniques for routine laboratory measurements. Some methods, such as atomic force microscopy (AFM)^4^ or the parallel plate rheometer^5^ have been successfully applied for investigation of the structural and physical properties, however they are limited in their ability to describe properties at acceptable resolution. For example, AFM-based nano-indentation of mature biofilms is limited to the examination of the biofilm-liquid interface region of a sample,^4^ whereas standard rheometer measurements describe the entire biofilm sample but provide only bulk average properties and, in most cases, require *ex situ *analysis and a quantity of biofilm sample that may not be practical.

Particle-tracking micro-rheology, which resolves the local mechanical properties spatially and temporally, has emerged as a promising technique to depict the biofilm at microscale.^6–10^ Birjiniuk et al. found that charge interactions play a key role in mobility within biofilms.^6^ Chew et al*.* discovered how genetic changes influence the biofilm mechanical property and structure formation. In *P. aeruginosa* biofilms, the role of known polysaccharides was elucidated: Psl increased the elasticity and effective cross-linking within the matrix while Pel reduced effective cross-linking within the matrix.^7^ Another study used magnetic tweezers via carboxylated magnetic bead probes embedded in biofilm. It was found that the creep compliance become higher with increasing height from the bottom of biofilm.^8^

Although each of these studies has provided insight into biofilm structure, there are few papers that quantitatively describe, for the same biofilm samples, both structural and mechanical properties *in* *situ*. This paper describes single particle tracking microrheology combined with confocal laser scanning microscopy to resolve the biofilm structure in three dimensions and calculate the creep compliances locally. Moreover, the biofilm was examined at different time scales (24 h and 48 h cultivation) and alternate environment conditions (with and without calcium chloride supplementation). The biofilm was analysed by classification into three layers (bottom, middle, top) depending on the normalized height and the tracking particles were segregated into two different biofilm regional characteristics (voids and clusters). This paper describes the heterogeneity of the biofilm structure and explains provides some data on the differences in material characteristics depending on cultivation time.

## Materials and methods

### Bacterial strains

The selected bacterial strain for this study was an mCherry-expressing *P. fluorescens* PCL1701,^11^ stored at –80 °C in King B broth^12^ supplemented with 20 % glycerol. Cultures were obtained by inoculating 100 ml of King B broth supplemented with gentamicin at a final concentration of 10 μg ml^−1^ using a single colony of a previously grown culture on King B agar (Sigma Aldrich, Ireland) at 28 °C. The inoculated medium was then incubated at 28 °C with shaking at 75 rpm and left to grow to late exponential growth phase, corresponding to an optical density (OD_600_) of about 1.0.

### Fluorescent bead preparation

Green fluorescent carboxylate micro beads (Sigma, L46) of 1 μm diameter were used in all biofilm microrheological experiments. A concentrated micro bead solution was first diluted (1:30) in Grade 1 quality water (18.2 MΩ cm^−1^) obtained from an Elga Process Water System (Biopure 15 and Pureflex 2, Veolia, Ireland), and will henceforth be referred to as MilliQ water. The suspension was then centrifuged at 10,000 RPM for 10 min. The supernatant was carefully discarded and the micro bead re-suspended with 25 μl volume of MilliQ. This sequence was repeated three times to remove any trace of surfactants from the solution the micro beads were provided in. Prior to biofilm growth experiments, micro bead pellets were re-suspended in sterile PBS-buffer solution.

### Biofilm growth and bead implantation

A 4 μl volume of an overnight culture of *P. fluorescens* was used to inoculate sterile individual centrifuge tubes (Falcon, Fischer Scientific, Ireland). Single autoclaved cover slips (Thermo Scientific, Germany, borosilicate glass 18 × 24 mm), were partially submerged into individual tubes. Biofilm that formed on these coverslips were the basis for all measurements in this study. Each tube contained an initial 3 ml of King B broth supplemented with gentamicin at a final concentration of 10 μg ml^−1^. Previously prepared green fluorescent polystyrene 1 μm diameter beads (Sigma, L46) were added into the King B medium to a final concentration of 5 × 10^5^ beads ml^−1^. To assess biofilm under an alternative ionic environment, tubes were supplemented with CaCl_2_ at a final concentration of 15 mM. Control tubes were not supplemented with CaCl_2_. Experiments were performed in triplicate with independently grown *P. fluorescens* culture incubated for 24 h or 48 h at 28 °C with shaking at 75 rpm.

### Confocal laser scanning microscopy

Following biofilm growth, coverslips were first rinsed in sterile PBS solution by gently dipping the coverslip containing biofilm in a tube containing sterile 0.1 M NaCl solution. The coverslip was then carefully placed in a single-well Nunc Lab-Tek II Chamber Slide (VWR, Ireland) filled with sterile phosphate buffered saline solution.

Horizontal plane images (xy-plane) of the biofilms were acquired using an Olympus FV1000 CLSM at the Live Cell Imaging core technology facility at the UCD Conway Institute. CLSM experiments were repeated three times for each biofilm growth condition using independently grown *P. fluorescens* cultures. At least three to four random areas were acquired for each biofilm sample per experiment. The excitation wavelength used for mCherry expressing *P. fluorescens* cells was 559 nm, and the emitted fluorescence was recorded within the range of 603 nm. The green fluorescent beads within biofilms were excited at 488 nm and emissions registered at 519 nm. Images were collected through an Olympus UPL SAPO 60 × NA:1.35 Oil objective with a z-step of 1 μm. Additionally, for each selected random position, time series of 2.25 s scanning time-increments were separately acquired from bottom to top regions within the biofilm depth, termed as z-horizontal planes, for approximately 135 s. Time series (xyt-) stacks were thereby composed of 60 slices and were analyzed to assess bead trajectory. 3D projections were performed using FIJI image processing tool’s 3D viewer plugin.

### Biofilm structure analysis

The structural quantification of biofilms was performed using the ISA3D MATLAB-based software developed by Beyenal et al*.*,^13^ allowing a detailed analysis of biofilm structural and textural properties. The latter includes textual energy, a parameter introduced by Haralick et al.^14^ as a measure of directionally repeating patterns of pixels in images. Lower textual energy values indicate less frequent and fewer repeated patterns of pixel clusters in one direction, and higher textual energy shows a more homogeneous image structure with fewer repeated patterns. The term entropy was first defined in communication theory by Shannon in 1948,^15^ and is a measure of uncertainty of information. Textural entropy in the context of biofilm samples as analysed by the ISA3D software represent the degree of randomness of pixels in the gray scale of image. Higher textual entropy values indicate increased levels of heterogeneity.

### Particle trajectory registration and Mean mean square displacement (MSD) analyses

Using the acquired CLSM xyt-time series stacks, particle trajectories were obtained using the Diatrack software (version 3.04 Pro).^16^ The obtained bead trajectories were used to calculate the MSDs as follows:

1 $${\rm{MSD = }}\left\langle {\Delta {r^2}\left( \tau \right)} \right\rangle = {\left\langle {r\left( {t + \tau } \right) - r\left( t \right)} \right\rangle ^2}$$

where *r* represents the position of a particle at time *t*, and *τ* is the lag time. In this study, the MSDs describe how embedded beads in *P. fluorescens* biofilms move within the biomass. These recorded migrations are then used to compute localized viscoelastic properties of the biofilm material, in the form of creep compliance, by recording the MSDs over time. The MSDs were computationally determined using a Matlab script, developed for the purpose of this study (cf. Fig. S1. supplementary material section).

The creep compliance, which is defined as the ratio of displacement to a given applied force over time, can be calculated from the MSDs. The MSD is therefore proportional to the creep compliance of the material in which the beads were embedded,^6^ and is expressed by the following equation:

2 $$J = \frac{{3\pi d}}{{4{k_B}T}}\left\langle {\Delta {r^2}\left( t \right)} \right\rangle$$

where *J* is the creep compliance, *d* is the diameter of the polystyrene beads, *t* is the temperature and *k*_*B*_ is the Boltzmann constant.

This method offers a non-destructive method of measuring the material properties of biofilms at different horizontal z-planes (bottom, middle and top). In combination with data obtained with confocal microscopy, the structural and mechanical properties of biofilms may be simultaneously assessed to provide an insightful analysis of the spatiotemporal development of biofilms.

### Developmental tool for the analysis of region-specific micro-rheology profiles

In order to classify beads into populations of confined and mobile populations based on obtained MSD data, an unbiased statistical model was used to group the two types of trajectories into two grouped populations. This is shown in supplementary Fig. S2 (cf. supplementary material section), where the product of the range [max(*r*_*x*_)−min(*r*_*x*_)] and the standard deviation *σ*_x_ of the x-coordinate of the particle. To determine an approximate cut-off, the particles that fall above the dashed line are designated as mobile while the ones below the cut off line were designated as immobile or confined.^17^ Following creep compliance measurements of all beads within a given biofilm field of view, contour maps were created using MINITAB v15.1 (Minitab Inc., State College, PA), by applying Akima's polynomial method for the interpolation of creep compliance values for each fluorescent bead’s x-y-coordinate.

### Statistical analysis

One-way analysis of variance (ANOVA) was performed using MINITAB v15.1 (Minitab Inc., State College, PA) in order to test the significant differences in biofilm structural parameters of *P. fluorescens* biofilms grown in the presence or absence of CaCl_2_ with Tukey’s test for pairwise comparisons. ANOVA was also performed to determine statistical differences in creep compliance (Pa^−1^) of different biofilm layers and regions (voids and cell clusters) of 24 h- and 48 h-grown *P. fluorescens* biofilms under the presence or absence of CaCl_2_. All tests were performed at the 5 % significance level.

## Results and discussion

### Biofilm structural and morphological properties

**Fig. 1:**
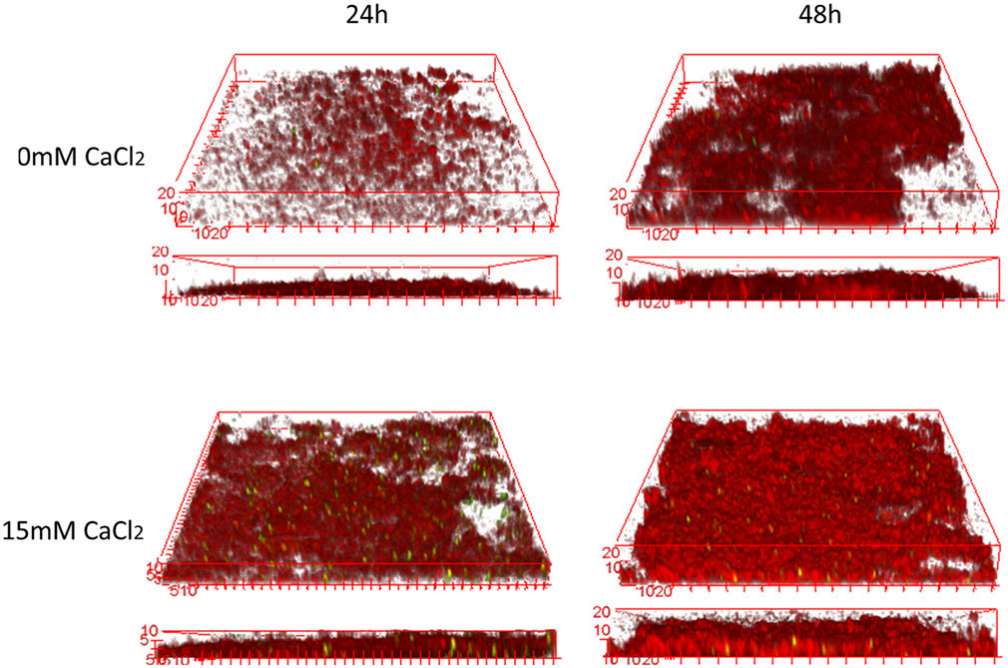
Three dimensional (xyz) and corresponding two dimensional (xz) CLSM projections of 24 h and 48 h mCherry-expressing *Pseudomonas fluorescens* following growth in the absence (0 mM) or presence (15 mM) of CaCl_2_. The green dots are fluorescent polystyrene beads (1 μm). 3D images were created with ImageJ’s “3D viewer” plugin

The effect of CaCl_2_ on *P. fluorescens* biofilm structure and morphology was first assessed over a two day period, as previously described by Safari et al.^4^ However, a higher magnification during CLSM acquisitions was used to locate and monitor embedded fluorescent beads within the biofilms matrix. Fig. 1 describes representative three dimensional projections of 24 h and 48 h old *Pseudomonas fluorescens* biofilms obtained following growth at 0 mM CaCl_2_ or 15 mM CaCl_2_ environments. Biofilms generally appeared to have increased in surface coverage from 24 h and 48 h growth periods. Biofilms grown in the absence of CaCl_2_ appeared to be less confluent as biofilms grown in the presence of CaCl_2_, hence confirming results previously obtained in a similar study.^4^ Biofilm cells also appeared to cover more surface area in the presence of CaCl_2_. Structural differences were however not easily distinguishable between biofilms grown under different CaCl_2_ environments as previously described.^4^ This limitation could be explained by the 63× lens used during this study, which allowed the acquisition of smaller biofilm areas with much higher magnification, as opposed to the 10× objective giving lower magnification and larger field of views. Although the qualitative analysis presented a number of limitations when comparing and interpreting acquired biofilms, the quantitative analyses of CLSM data using Image structure analysis tool allowed an in-depth study of the structural and textural biofilm properties in this study (Table 1AB). Structural biofilm properties such as biovolume and mean thickness increased, while decreasing in porosity and biofilm roughness parameters from 24 h to 48 h growth periods (Table 1A). However, these changes were insignificant (*p* > 0.05) in the absence of CaCl_2_. Changes in biofilm structure was found to be significant in the presence of CaCl_2_ from 24 to 48 h development for parameters such as biovolume (*p* = 0.003), mean thickness (*p* = 0.022), and biofilm roughness (*p* = 0.046), while the drop in biofilm porosity was insignificant (*p* = 0.29).

**Table 1:** Biofilm structural (A) and textural (B) properties

A.								
	Structural biofilm parameters							
	Biovolume (μm^3^)		Porosity		Mean Thickness (μm)		Biofilm roughness	
	24 h	48 h	24 h	48 h	24 h	48 h	24 h	48 h
0 mM CaCl_2_	8.4 × 10^4^ ± 2.0 × 10^4^	1.4 × 10^5^ ± 3.3 × 10^4^	0.90 ± 0.02	0.88 ± 0.02	4.33 ± 1.41	7.54 ± 2.49	1.19 ± 0.11	0.97 ± 0.07
15 mM CaCl_2_	1.5 × 10^5^ ± 1.7 × 10^4^	2.2 × 10^5^ ± 9.9 × 10^3^	0.83 ± 0.02	0.80 ± 0.02	6.2 ± 0.88	9.25 ± 0.84	0.76 ± 0.06	0.61 ± 0.07

B.						
	Textural biofilm parameters					
	Textural entropy		Energy		Homogeneity	
	24 h	48 h	24 h	48 h	24 h	48 h
0 mM CaCl_2_	4.46 ± 0.54	5.36 ± 0.42	0.20 ± 0.08	0.07 ± 0.02	0.64 ± 0.05	0.55 ± 0.04
15 mM CaCl_2_	6.43 ± 0.31	7.30 ± 0.49	0.02 ± 0.01	0.02 ± 0.01	0.45 ± 0.03	0.37 ± 0.05

When comparing the effect of CaCl_2_ on both 24-h and 48-h *P. fluorescens* biofilms, high calcium concentrations led to significant changes to structural and textural properties. The presence of CaCl_2_ contributed to increased biovolume (*p* < 0.05), lower biofilm porosity (*p* < 0.05) and roughness (*p* < 0.05). Mean thickness was shown to not have been significantly affected by the presence of CaCl_2_ in both 24 h-and 48 h old biofilms with *p* values of 0.293 and 0.523, respectively.

Biofilm textural analysis (Table 1B) revealed that the presence of CaCl_2_ led to changes in the form of biofilm heterogeneity. This was particularly apparent by the increased textural entropy and lowered homogeneity parameters compared to growth in the absence of CaCl_2_. These results confirm the qualitative observations obtained in a previous study in which the presence of calcium led to an increase in global roughness.^4^ This heterogeneity was also determined by the energy parameter,^13^ which was found to be generally lower in the presence of CaCl_2_, indicating a higher frequency of repeating cluster patters, and therefore a more heterogeneous biofilm.

**Fig. 2:**
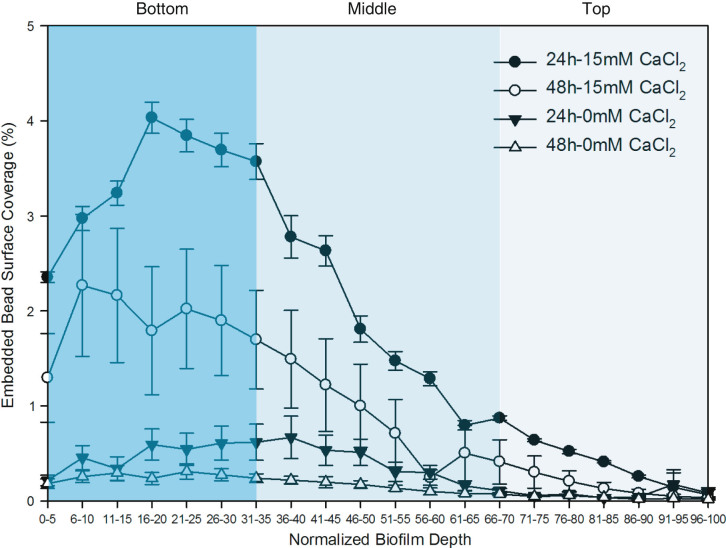
Spatial stratification of embedded fluorescent 1 μm beads presented as surface coverage (%) vs. biofilm depth following 24 h, 48 h biofilm development in the absence and presence of CaCl_2_. Error bars represent standard error of the mean

Biofilms were grown in the presence of embedded fluorescent nanoparticles, with the objective of purposely analysing for subsequent microrheological studies. The advantage with this approach was the ability to measure beads throughout the biofilm depth, thereby providing a detailed viscoelastic characterization profile in a non-destructive *in situ* manner, as opposed to the traditional destructive classical rheology and force spectroscopy techniques. The beads in this study were uniformly embedded in the biofilms and their surface coverage throughout biofilm depth is presented in Fig. 2. The bead surface coverage parameter at various depths was found to be influenced by biofilm age (*p* = 0.018) and exposure to CaCl_2_ (*p* < 0.001) during growth. In terms of bead localization, results showed that beads embedded in biofilm grown in the absence of CaCl_2_ were generally equally spread in the bottom and middle layers of the biofilm. The bead profile of biofilms grown in the presence of CaCl_2_ revealed a gradual decrease in bead surface coverage from bottom to top biofilm layers with the highest surface coverage at the bottom regions of the biofilms. These results suggest that the level of bead interaction during biofilm development is not only cultivation-time dependent but is also linked to the environment at the time of these interactions take place. The higher level of bead surface coverage within biofilms could have been attributed to the higher adhesive nature of *P. fluorescens* biofilms in the presence of CaCl_2_ as recently described,^4^ thereby promoting bead-biofilm interactions. The higher level of beads at the bottom layers also suggests that this interaction may have initially occurred at the onset of biofilm development. Interestingly, the decreasing bead surface coverage from bottom to top biofilm layers is indicative of possible variations in mechanical, and viscoelastic internal biofilm properties which can be better understood through microrheological techniques. A non-destructive *in situ* approach such described in this study is therefore useful in understanding potential mechanical and viscoelastic variation within the entire biofilm depth.

### Micro-rheological analysis of biofilms

#### MSD

The mechanical properties of 24 h and 48 h biofilms formed in the presence or absence of CaCl_2_ were analysed by following the MSDs and creep compliance of 1 μm polystyrene beads embedded different biofilm depths and regions. Biofilm layers were classified into bottom, middle and top zones according to normalized biofilm depths of 0–33, 34–66 and 67–100, respectively. Vibrations from embedded 1.0 μm beads at fixed positions were recorded and the analysed MSD values were typical of viscoelastic materials.

**Fig. 3:**
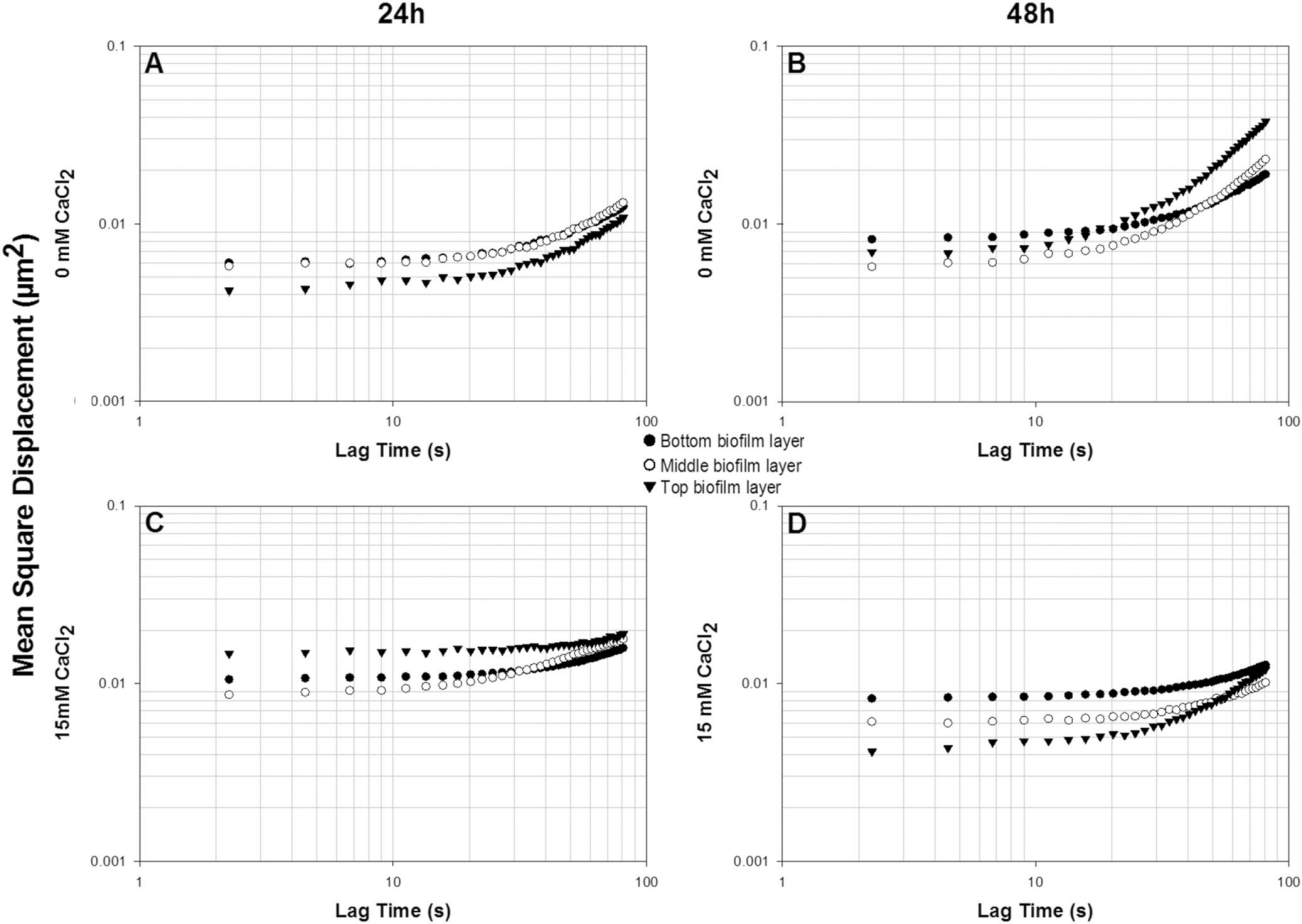
MSD profiles of 1 μm fluorescent beads within bottom, middle and top biofilm layers of 24 h (**a**, **c**) and 48 h (**b**, **d**) *Pseudomonas fluorescens* biofilms grown at 0 mM (**a**, **b**) and 15 mM (**c**, **d**) CaCl_2_ environments

The MSDs of all tested biofilms (Figs. 3a, b, c, d) exhibited a viscoelastic profile based on the fitting of MSDs∝*t* ^*α*^. The calculated exponent α is superior to 0 (elastic) and 1 (viscous/liquid) (cf. Table S1. supplementary material section). Interestingly, MSD values indicated distinct rheological differences within biofilm layers over time depending on the salt environment in which the biofilms were grown.

In the absence of CaCl_2_ (Fig. 3a, b), the median MSDs ranged between 6.5 × 10^−3^ to 8.26 × 10^−3^ μm^2^ within 24 h biofilms (Fig. 3a) to MSD values between 1.6 × 10^−2^ and 1.2 × 10^−2^ μm^2^ within 48 h biofilms (Fig. 3b). This increase in MSD values within 48 h biofilms is an indicator of the increased ductile nature of the biofilm, through the loosening of biofilm matrix allowing greater bead displacement. Detailed MSD analysis of the different biofilm layers revealed that the top layer of 24 h biofilms grown in the absence of CaCl_2_ went from being the least malleable to the most malleable layer following 48 h growth. This change, observed by the increase MSD values, suggests that the rheological profile of the biofilm matrix is biofilm depth dependent, and may be linked to the changes in the chemical microenvironments associated with biofilm growth.^18, 19^

In the presence of CaCl_2_ (Fig. 3c, d), the median MSDs ranged between 1.6 × 10^−2^ to 1.2 × 10^−2^ μm^2^ within 24 h biofilms (Fig. 3c) to MSD values between 6.9 × 10^−3^ and 9.8 × 10^−3^ μm^2^ within 48 h biofilms (Fig. 3b). This decrease in MSDs between 24 h and 48 h growth periods is indicative of a generalised stiffening of the biofilm, through the increase in effective cross-linking in the matrix network within biofilms caused by the presence of calcium, known for its crosslinking and gel stiffening properties. Interestingly, the rheological properties of top biofilm layer went from being most malleable following 24 h to being the stiffest biofilm layer following 48 h development.

Comparing biofilms grown at different cultivation times, the presence of CaCl_2_ (Fig. 3c) generally led to more ductile biofilms following 24 h (Fig. 3a). Following longer growth periods, the presence of CaCl_2_ (Fig. 3d) led to a stiffer biofilm compared to growth in the absence of CaCl_2_ (Fig. 3b). These differences could be associated with the structure and maturity of biofilms as previously described. The stiffer biofilm in the absence of calcium following 24 h could be explained by the higher ratio of cells to EPS.^4^ Interestingly, after 48 h, biofilms grown under calcium supplementation (Fig. 3d) were much stiffer than biofilm development without CaCl_2_ (Fig. 3c). Moreover, the rheological profile through the biofilm depth suggests that calcium led to stiffer top layers.

#### Creep Compliance

**Fig. 4:**
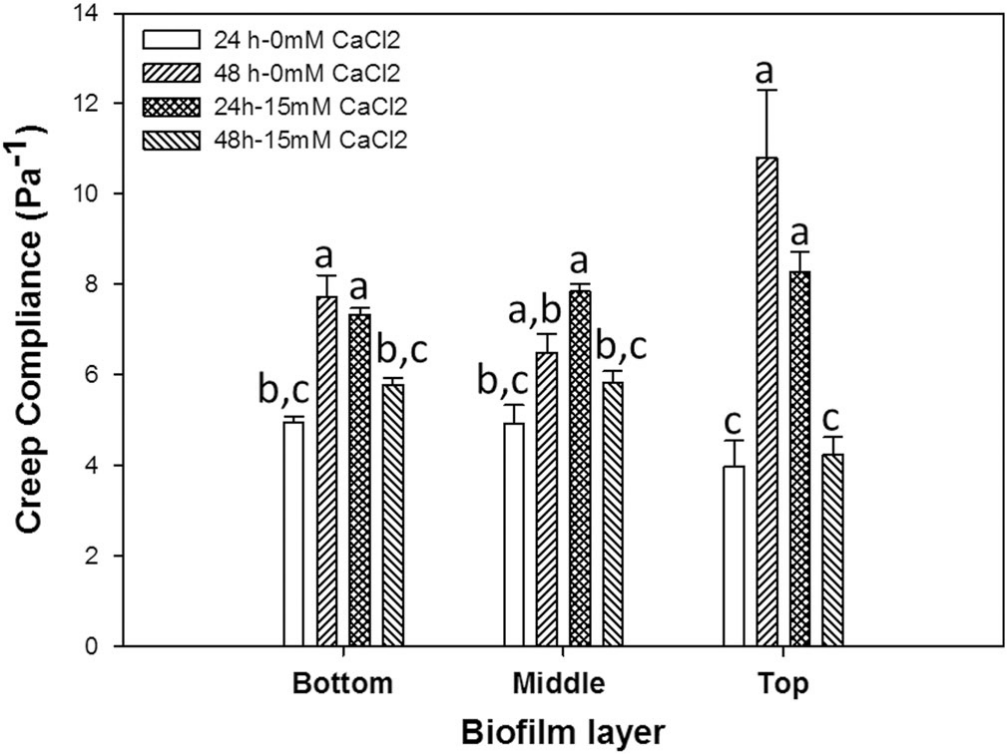
Mean creep compliance of *Pseudomonas fluorescens* bottom, middle and top biofilm layers following 24 h and 48 h-biofilm development under the absence and presence of CaCl_2_ environments. Means that do not share a letter are significantly different. Error bars represent standard error of the mean

Creep compliance, which is a measurement of how materials deform over time, was calculated based on the MSD values for all biofilm samples to example the effect of cultivation time and CaCl_2_ concentration (Fig. 4). The analysis presented here allows quantification of the rheological properties in different layers within the same biofilm sample. Contrarily to what was observed with the MSD data, there were no statistical differences (*p* > 0.05) between bottom, middle and top layers for biofilms grown in their respective conditions. It should however be noted that the lack of creep compliance variations between the layers is separate to the heterogeneous and complex nature of the biofilm. The use of beads for the microrheological studies of biofilms should therefore not be limited to a layered biofilm classification, but rather, should selectively include regions within the biofilm. Since biofilms are structurally heterogeneous, MSDs and creep compliance data analysis should take this into consideration. A modified approach was therefore developed to analyse MSDS and creep compliance of key biofilm regions to investigate region specific aspects of biofilm micro-rheology*.* Koza et al. (2009) studied the viscoelastic properties of *P. fluorescens* SBW25 biofilms cultivated at the air-liquid interface by using a parallel plate rheometer. The stress amplitude sweep tests were conducted at 0.5 Hz, yielding a shear modulus of 0.75 Pa, which is close to what was observed in this present study.^20^ This proves that the non-destructive microrheological method described in this study is in good agreement with the classical yet destructive rheological methods (i.e., parallel plate rheometer) for the quantification of biofilm mechanical properties.

### Micro-rheological analysis of biofilm regions of interest

**Fig. 5:**
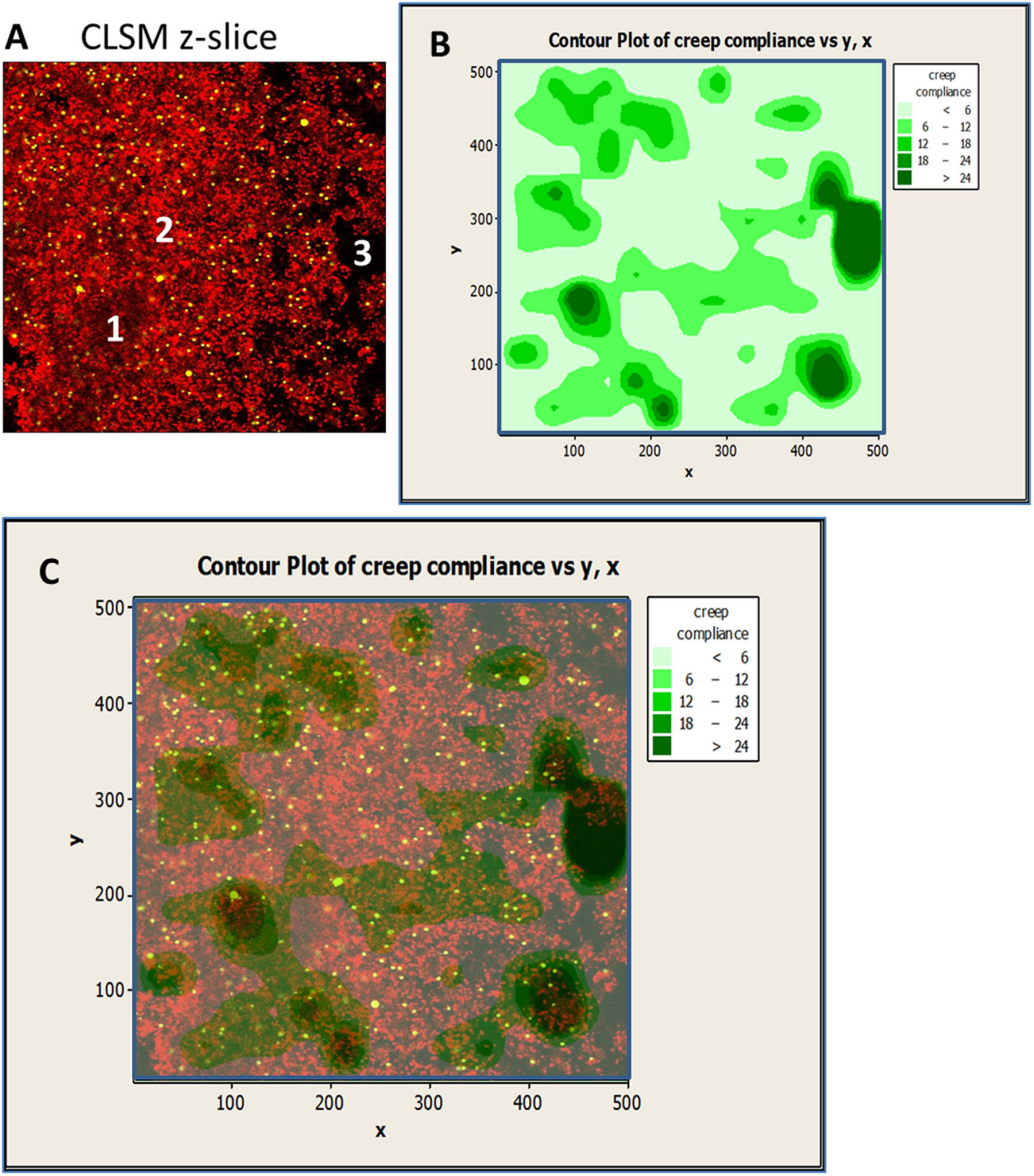
Region-specific Creep compliance analysis. **a** Example of a horizontal plane (xy-) micrograph of a biofilm layer depicting mCherry-expressing *P. fluoresence* cells (*red*) and 1 μm fluorescent beads (*green*). Zones 1, 2 identify biofilm areas of high low cell density and high cell density respectively. Zone 3 is recognized as surrounding bulk suspension. **b** Bead trajectories from zones 1 and 2 are used to calculate MSDs used to determine Creep compliance. Akima's polynomial method for the interpolation of creep compliance values for each fluorescent bead’s xy-coordinate was used to generate contour maps. **c** By merging xy-micrographs and corresponding contour maps, region-specific creep compliance analysis is possible

Upon further examination of embedded beads within biofilm depths, beads were found to be localized within specific regions characterized as biofilm voids (zone I) and clusters (zone 2) (Fig. 5). Biofilm voids in this study were defined as areas with distinctively lower mCherry fluorescence and opaque in nature compared to surrounding bulk liquid areas (zone 3), while areas of high cell density characterized by the expression of mCherry fluorescent protein depicted as area 2 (Fig. 5a). Void and bulk liquid areas were differentiated by their distinct bead trajectories within these zones as illustrated in supplementary Fig. S3.

**Fig. 6:**
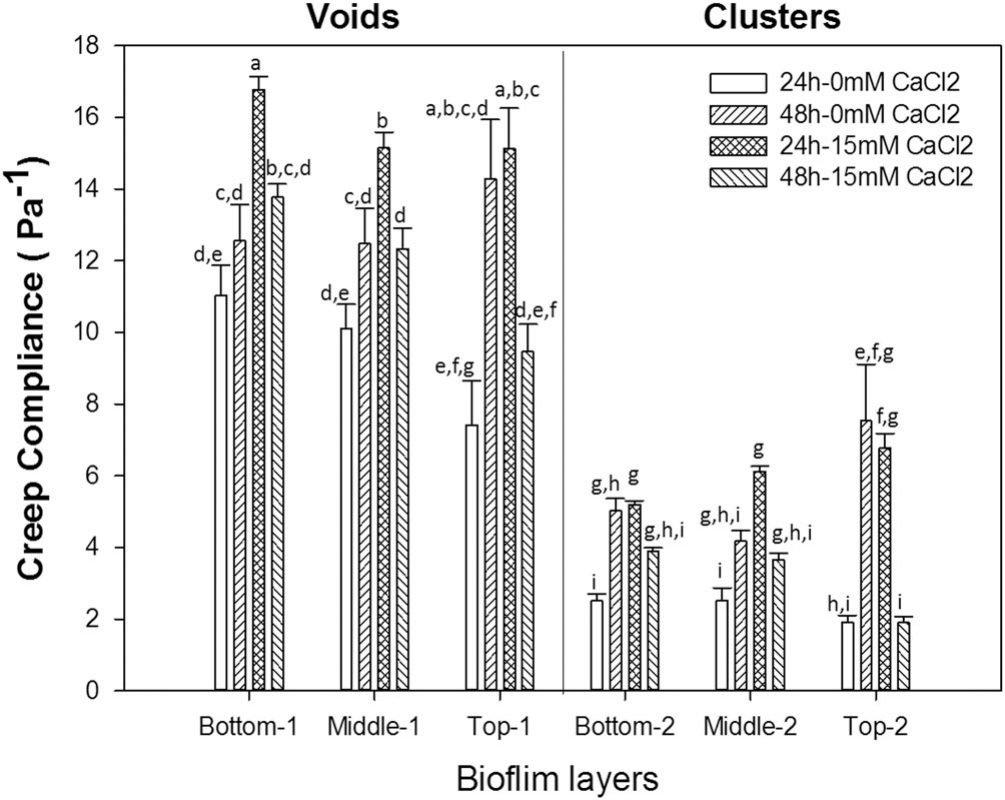
Mean creep compliance of *Pseudomonas fluorescens* void and cell cluster regions within bottom, middle and top biofilm layers following 24 h and 48 h-biofilm development under the absence and presence of CaCl_2_ environments. Means that do not share a letter are significantly different. Error bars represent standard error of the mean

The MSD analysis of fluorescent beads within biofilm layers was repeated, however this time by taking into consideration the spatial heterogeneities in each layer. By plotting the trajectories of each recorded bead within their respective field of view and biofilm layers, contour maps of corresponding creep compliance values were generated, delimitating zones of high and low ductility (Fig. 5b). These contour maps revealed that the rheological profile within the different layers were highly heterogeneous with areas of high ductility described by high creep compliance range (dark green) to stiffer zones of lower creep compliance values (light green). By overlapping the generated contour plots with corresponding CLSM micrographs (Fig. 5c) it was revealed that the void areas were characterized by high creep compliance values, while cluster areas were defined with lowest creep compliance. These results seem to suggest that the voids areas may be composed of a significant amount of exopolymeric substances compared to cluster areas. While void areas were distinctively composed of lower cell density (based on mCherry fluorescence), their high ductility can thus be attributed to low cell-to-EPS ratios. Cluster area having higher cell densities and therefore higher cell-to-EPS ratios was shown to be generally stiffer. These results add weight to the conclusions of a hypothesis described in a recent study where the cell-to-EPS ratios was found to influence the viscoelasticity of biofilms.^4^ While Safari et al*.* ^4^ assessed the viscoelastic properties of 5 % of the uppermost biofilm top layer, the force spectroscopy employed in that study was restricted by its limited (by depth) range of biofilm sampling. The microrheological method presented in this study goes beyond the limitations of force spectroscopy, and offers the potential for an improved understanding of region-specific material property characteristics at different biofilm layers. This was achieved by comparing the regional-based creep compliance values of the different biofilm layers as shown in Fig. 6.

The region-based creep compliance analysis led to the rheological differentiation of clusters and void regions (*p* < 0.0001), where the former was found to be generally stiffer than void areas irrespective of biofilm layer or biofilm growth conditions and growth period (cf. Fig. S3 in the supplementary material section). Similar profiles were observed by Birjiniuk et al*.*, in which embedded beads within biofilms were separated by their level of mobility and confinement.^6^ Interestingly the rheological dynamics profiles within voids and clusters showed similar trends to global rheological trends presented in Fig. 4. Moreover, results from regional creep compliance analysis also suggest that the creep compliance of biofilm void zones is the primary contributor to biofilm mechanical properties, contributing to the overall viscoelastic character. The low creep compliance profile of cluster areas may be linked to the higher density of cells there.

## Conclusions

The use of 1 μm beads for the purpose of microrheological measurements combined with confocal imaging of biofilm structure was successful. MSD measurements were used to determine biofilm creep compliances, which allowed visualisation of region-specific microrheological profiles within the biofilm. Compared to force spectroscopy or classical (bulk) rheological techniques, the micro-rheological method described in this study offers the possibility of investigating region-specific biofilm rheological properties in an *in-situ* and non-destructive manner. Future improvements to this technique could include localised chemical analysis of biofilms to determine whether the EPS within regions of distinct creep compliance profiles is related to either the type of EPS present in those regions or the degree of unrestricted displacement based on cell density. With the unmet need to control unwanted biofilms, understanding biofilm micro-rheological profiles is expected to become a crucial step in optimizing novel biofilm treatments and eradication strategies.

## Electronic supplementary material

Supplementary Table

Supplementary Information

Supplementary Details

Supplementary Details
