# Supplementary material for: Revealing region-specific biofilm viscoelastic properties by means of a micro-rheological approach
Source: NPJ Biofilms Microbiomes. 2016 Dec 5;2:5. doi: 10.1038/s41522-016-0005-y (PMC5460257; doi:10.1038/s41522-016-0005-y)
Supplement: Supplementary file 1 — Supplementary Table [file 41522_2016_5_MOESM1_ESM.docx]

**Table S1:** The exponent $\alpha$ fitting values of different groups

|  | 0mM CaCl_2_ 24h | 0mM CaCl_2_ 24h | 15mM CaCl_2_ 24h | 15mM CaCl_2_ 48h |
| --- | --- | --- | --- | --- |
| Top layer | 0.296 | 0.580 | 0.069 | 0.334 |
| Mid layer | 0.260 | 0.455 | 0.246 | 0.163 |
| Bottom layer | 0.238 | 0.271 | 0.125 | 0.134 |
