# Supplementary material for: Revealing region-specific biofilm viscoelastic properties by means of a micro-rheological approach
Source: NPJ Biofilms Microbiomes. 2016 Dec 5;2:5. doi: 10.1038/s41522-016-0005-y (PMC5460257; doi:10.1038/s41522-016-0005-y)
Supplement: Supplementary file 2 — Supplementary Information [file 41522_2016_5_MOESM2_ESM.pdf]

```
clc
num = xlsread('biofilm+beads48hA-xyt-5micron.xlsx');
bead_num = max(num(:,2));
num_images=max(num(:,3))+1;
X=zeros(num_images,bead_num);
Y=zeros(num_images,bead_num);
Z=zeros(num_images,bead_num);
for j=1:bead_num
    for k=1:num_images
        X(k,j)=num((num_images*(j-1)+k),4);
        Y(k,j)=num((num_images*(j-1)+k),5);
        Z(k,j)=num((num_images*(j-1)+k),6);
    end
end
%MSD calc
MSDgrid=nan(num_images-1,bead_num);
for i= 1:bead_num
MSDdist=nan(num_images-1, num_images-1);
for j= 1: num_images-1
    if X(j,i)>1
for k= 1: num_images-j
    if X(j+k,i)>1
    MSDdist(j,k)=(((X(j,i)-X(j+k,i))*0.414)^2)+(((Y(j,i)-Y(j+k,i))*0.414)^2);
    end
end
    end
end
MSDgrid(:,i)=nanmean(MSDdist);
end
figure(1)
clf
plot(MSDgrid)
grid on
figure(2)
clf
avMSDgrid=nanmean(MSDgrid,2);
plot(avMSDgrid)
grid on
```
