# Supplementary figures and images for: Revealing region-specific biofilm viscoelastic properties by means of a micro-rheological approach
Source: NPJ Biofilms Microbiomes. 2016 Dec 5;2:5. doi: 10.1038/s41522-016-0005-y (PMC5460257; doi:10.1038/s41522-016-0005-y)

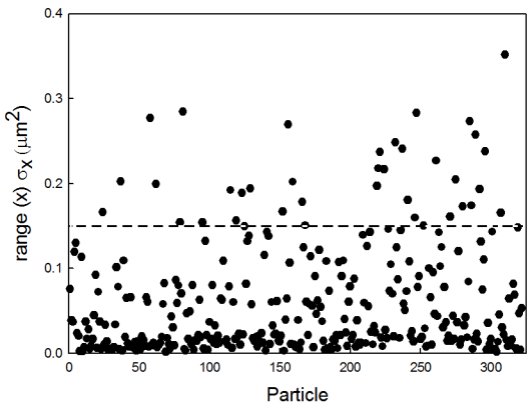

Supplement: Supplementary file 3 — Supplementary Details [file 41522_2016_5_MOESM3_ESM.pdf]

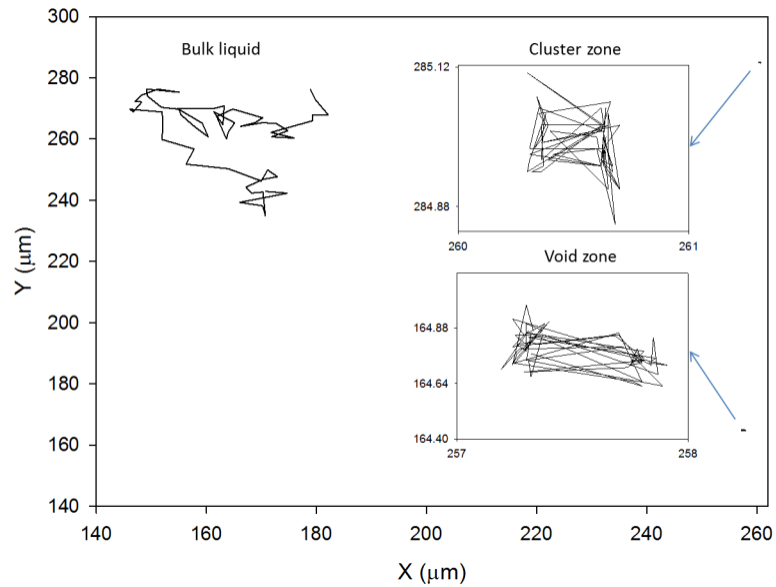

Supplement: Supplementary file 4 — Supplementary Details [file 41522_2016_5_MOESM4_ESM.pdf]
